# Phytochemical and Antioxidant Studies on a Rare *Rheum cordatum* Losinsk. Species from Kazakhstan

**DOI:** 10.1155/2019/5465463

**Published:** 2019-11-16

**Authors:** Gulsim Zhumashova, Wirginia Kukula-Koch, Wojciech Koch, Tomasz Baj, Galiya Sayakova, Alma Shukirbekova, Kazimierz Głowniak, Zuriyadda Sakipova

**Affiliations:** ^1^School of Pharmacy, S.D. Asfendiyarov Kazakh National Medical University, Tole-bi 94, 050012 Almaty, Kazakhstan; ^2^Department of Pharmacognosy, Medical University of Lublin, 1 Chodzki Str., 20-093 Lublin, Poland; ^3^Department of Food and Nutrition, Medical University of Lublin, 4a Chodzki Str., 20-093 Lublin, Poland; ^4^Department of Pharmaceutical Disciplines, Astana Medical University, Beybitshilik 49A, 010000 Nur-Sultan, Kazakhstan; ^5^Department of Cosmetology, University of Information Technology and Management in Rzeszów, Kielnarowa 386a, 36-020 Tyczyn, Poland

## Abstract

An optimisation of extraction towards an increased antioxidant capacity and the study on the extracts' composition by HPLC-ESI-Q-TOF-MS were performed on different organs of a rarely studied plant: *Rheum cordatum* Losinsk (Polygonaceae) growing in Kazakhstan. More than 20 compounds from anthraquinones and phenolics were identified in an optimised method. The plant was proven to contain a wide variety of phenolic compounds (catechins, flavonoids, and their glucosides and phenolic acids) in contrast to the anthraquinone composition, which was mainly represented by emodin and its analogues. The results of the studies could determine the plant as a rich source of pharmacologically precious polyphenols. It was evidenced that the extracting solvents, the time of collection, and the organs tested affected both the chemical content and the antioxidant potential of the extracts. Ethanol : water (50 : 50 *v*/*v*) was selected as the most beneficial extractant for all metabolites, and based on the principal component analysis of raw data, the radical scavenging potential of the plant was strictly related to the presence of epicatechin gallate (ECG), kaempferol glucoside, and epigallocatechin gallate (EGCG) occurring in this extract at the concentration of 1.69-5%, 0.16-0.47%, and 0.001-2.93%, respectively.

## 1. Introduction

The genus *Rheum* (rhubarb) belongs to the family Polygonaceae and contains about 50 species of perennial herbaceous plants with a strong root system that are widely distributed and cultivated in Asia and Europe. The most known species, *Rheum tanguticum*, *R. palmatum*, and *R. officinale*, find their place in the pharmacopoeial monographs of Europe and Asia [1–5].

Rhubarbs belong to plant medicines known as far back as 2700 BC. Chinese traditional medicine used the rhubarb root for medicinal purposes to stimulate appetite, induce bile production, and treat hepatitis, intestinal atony, and flatulence. In large doses, the plant was administered as a laxative agent, and in powdered form, it was used externally as a wound-healing drug [4–6]. According to the literature data, anthraquinones such as emodin, aloe-emodin, rein, and chrysophanol are responsible for their laxative effect. Anthrons, stilbenes, flavonoids, acylglucosides, and pyranones, identified in a variable quantity, were found responsible for its antioxidant, hepatoprotective, antimutagenic, or antibacterial action [7–13].

Kazakhstan is known as a homeland of rhubarbs. As many as nine species grow on its territory and have been used for centuries in the traditional medicine of the country. The phytochemical study of the main groups of biologically active substances of Kazakhstani rhubarbs was held in the middle of the last century by the group of Professor T.K. Chumbalov. In his research, he evaluated the content of flavonoids in three of the most common Kazakhstan species of the genus *Rheum* (*Rheum cordatum* Losinsk, *Rheum tataricum* L., and *Rheum wittrockii* Lundstr.). The preliminary studies showed the highest potential of the heart-shaped rhubarb (*Rheum cordatum* Losinsk.). Based on these findings, the authors decided to develop the studies on the phytochemical characterization and antioxidant potential of the plant using modern analytical techniques, especially considering the initial studies of Professor Chumbalov, who identified catechins in the roots of *Rheum cordatum* and *Rheum wittrockii* [14–17]. Also, the authors' intention was to perform a careful identification of the secondary metabolites with possible antioxidant potential, in all organs of the species and an analysis of a seasonal diversity of anthraquinone derivatives and phenolics in the roots of the plant, by means of LC-MS instrumentation.

## 2. Materials and Methods

### 2.1. Plant Material

In the territory of Kazakhstan, the heart-shaped rhubarb grows in three floristic areas: along gravelly and stony slopes of Chu-Ili Mountains, Karatau, and Western Tien Shan [17, 18]. The extracts investigated in the study were obtained from roots, leaves, seeds, and petioles (stems) of *Rheum cordatum* Losinsk., collected in the Chu-Ili mountains, in the Kurday Pass, Zhambyl region, Kazakhstan, in October 2017 (roots, seeds), April 2018 (roots, petioles, and leaves), and May 2018 (roots). They were authenticated at the Institute of Botany and Phytointroduction, Almaty, Kazakhstan, by the general director doctor of biological sciences G. Sitpayeva. The samples of dried plant material are kept in the Department of Pharmacognosy with Medicinal Plants Unit at the Medical University of Lublin, Poland, under the numbers WKK18001 to WKK18006.

### 2.2. Chemicals and Reagents

2,2-Diphenyl-1-picrylhydrazyl (DPPH), Folin-Ciocalteu reagent, and gallic acid were purchased from Sigma-Aldrich. LC-MS spectroscopic-use reagents, methanol, water, hydrochloric acid, and formic acid, were manufactured by Merck (Darmstadt, Germany).

The solvents used for antioxidant assay and TPC measurements were of reagent grade and were purchased from Avantor Performance Materials (Gliwice, Poland). The standards of rutoside, ECG, and isticine were used for the preparation of calibration curves suitable for the quantitative analyses. The standards of other catechins (catechin (CA), epicatechin (ECA), and EGCG), quercetin, kaempferol, and apigenin were used for the identification purposes. All of them were purchased in Merck (Darmstadt, Germany) and their purity exceeded 95%.

### 2.3. Extraction

5 g of dried and finely powdered organs of *Rheum cordatum* was extracted in 20 mL of different solvents (ethanol, water, ethanol : water 50 : 50 *v*/*v*, chloroform, and dichloromethane) three times, 30 min each, on an ultrasonic bath at room temperature. The extracts were later centrifuged at 3500 rpm for 10 min and the supernatant collected, transferred to weighted Eppendorf vials, and evaporated at 45°C using an Eppendorf Concentrator Plus (Hamburg, Germany). The obtained extracts were refrigerated at 4°C and used for the bioactivity assessment and chromatographic analyses.

### 2.4. LC-ESI-Q-TOF-MS Analysis of the Obtained Extracts

The chromatographic analyses were performed using a 6500 Series LC-ESI-Q-TOF-MS system (Agilent Technologies, Santa Clara, CA, USA) equipped with an autosampler (G1329B), a degasser (G1322A), a DAD detector (G1315D), and a binary pump (G1312C). The identification of active constituents was performed using a Q-TOF mass spectrometer (G6530B) also by Agilent Technologies (Santa Clara, CA, USA). The analytes were separated on a 150 mm long Zorbax RP 18 column (Agilent Technologies, Santa Clara, CA, USA) (width: 2.1 mm, dp = 3.5 *μ*m) at a flow rate of 0.2 mL min^−1^ and a temperature of 25°C. The injection volume was set at 20 *μ*L. The mobile phase was composed of 0.1% formic acid (solvent A) and 0.1% of formic acid in acetonitrile (solvent B). The gradient elution was as follows: 0 min: 98% A; 5 min: 90% A; 20 min: 60% A; 35 min: 5% A; and 36 min: 98% A, whereas the analysis time was equal to 45 min and the flow rate to 0.2 mL/min. The DAD detector continuously recorded the absorbance from 190 to 600 nm, and later, the chromatograms were analysed at 254, 280, 320, and 365 nm. Mass spectra were simultaneously acquired in both negative and positive ionization modes, and the *m*/*z* signals were recorded in the range of 100-1200 using a capillary voltage of 3.5 kV, at gas and sheath gas temperatures of 350 and 325°C, respectively, gas flows of 12 L/min, the skimmer voltage of 65 V, the fragmentation voltage of 150 V, and the CID values of 10 and 20 V. The MS/MS spectra were recorded for the two most intensive peaks each time. The *m*/*z* signals with a single MS/MS spectrum recorded were excluded for the following 0.2 min to provide the fragmentation of less abundant signals. The quantitative analysis was performed based on the fragmentation spectra, literature data, and retention times.

### 2.5. The Antioxidant Activity of the Investigated Samples

#### 2.5.1. DPPH Test

All extracts were evaluated for their ability to quench free radicals using a 1,1-diphenyl-2-picrylhydrazyl (DPPH) assay according to a modified method described elsewhere [18]. The antioxidant activity was expressed as a percent of radicals' inhibition and was compared with gallic acid. Briefly, two concentrations of each extract were selected to determine their antioxidant potential: 1000 and 500 *μ*g/mL. The extracts dissolved in DMSO at a quantity of 0.1 mL were mixed with 1.9 mL of ethanolic solution of DPPH prepared immediately before the test at a concentration of 6 mg/100 mL. The solutions were mixed and stored in the dark for 30 min. Afterwards, the absorbance of the solutions was measured in 515 nm using a Unicam Helios Gamma (Thermo Electron Corporation) spectrophotometer. The solution of 0.1 mg/10 mL of gallic acid was used as a reference [19]. The measurements were repeated 6 times for each sample.

#### 2.5.2. Total Phenolic Content (TPC)

The same extracts were studied in a modified protocol for the Folin-Ciocalteu total phenolic content determination [20, 21]. The obtained results were expressed as gallic acid equivalents (GAE). For this purpose, a calibration curve of gallic acid was prepared for several concentrations of the reagent: 25, 50, 75, 100, 200, 250, 300, 350, 400, and 800 *μ*g/mL in DMSO. 0.5 mL of each gallic acid solution or extract at a concentration of 500 *μ*g/mL was mixed with 2.5 mL of a diluted Folin-Ciocalteu reagent (0.25 mL of Folin-Ciocalteu reagent with 4.25 mL of distilled water) in dark glass vials. Next, 2 mL of 7.5% solution of sodium carbonate was added to the samples and the absorbance was measured after 30 min at 765 nm. The absorbance of the tested extracts was used to determine their number of gallic acid equivalents from the sketched gallic acid calibration curve. All experiments were repeated 6 times for each extract. The statistical elaboration of the results was performed using Statistica 10.0 PL Software.

### 2.6. Statistical Analysis of Results

All extracts were obtained in triplicate and divided into either antioxidant analyses or the LC-MS-based determination of composition and quantity. Six injections were programmed for each extract on the LC-MS apparatus to provide sufficient quantity of data for further processing. Statistical analysis was performed using the Statistica 12.0 program. The significance of the results was tested by the one-way ANOVA method for *p* < 0.05. The matrix for grouping objects and features was carried out after prior standardization of the results using the *Z*-score method. Principal component analysis (PCA) was performed for all determined compounds showing a statistically significant correlation between their quantity and antiradical activity of the extracts (*p* < 0.05).

## 3. Results and Discussion

According to Baitenov [22], nine rhubarb species are spread on the territory of Kazakhstan, seven of which are characterized by medicinal properties. These are the heart-shaped rhubarb (*R. cordatum*), compact rhubarb (*R. compactum*), Wittrock rhubarb (*Rheum wittrockii*), Maximovicz rhubarb (*R. maximowiczii*), Tatar rhubarb (*R. tataricum*), low rhubarb (*R. nanum*), and Turkestan rhubarb (*R. turkestanicum*). These plants are administered to patients in the form of broths, infusions, alcoholic extracts, externally used powders, milk decoctions, but also marmalades, sodas, or compotes [22, 23]. The herein investigated heart-shaped rhubarb (*Rheum cordatum* Losinsk.) has been implemented in the treatment of digestive system diseases: stomach disorders and peptic ulcers; however, the studies on its components are scarce.

### 3.1. The LC-ESI-Q-TOF-MS Qualitative Analysis of the Extracts

In the current study, a careful profiling of *R. cordatum* extracts was achieved thanks to the application of hyphenated techniques characterized by a high sensitivity and high mass accuracy measurements. The application of the HPLC-ESI-Q-TOF-MS instrument in both positive and negative ionisation modes and in different fragmentation and collision energies provided clear chromatograms and informative fragmentation spectra. The identification of the major constituents of the extracts was performed in the negative ionisation mode, which was found to contain a larger number of signals in contrast to the positive mode. Similar to other authors, anthraquinones and single phenolics were determined in the negative ionisation mode [24]. Only flavonoids were visible in both positive and negative ionisation modes, which was helpful in the identification of this group of secondary metabolites. As apigenin and their derivatives are of the same molecular formula and theoretical mass as emodin and aloe-emodin, the analysis of the positive ionisation mode spectra was helpful in the differentiation of these two groups of secondary metabolites.

Having studied the scientific literature, and compared the previously identified compounds in other rhubarbs, the authors noticed that the *Rheum cordatum* belongs to the plants with a higher content of phenolic metabolites from anthraquinones. In all analysed extracts, the authors distinguished these two groups of components as the major ones (see Table 1). High resolution MS/MS measurements recorded with the energy of 10 or 20 V provided reliable data to study the fragmentation patterns of the majority of the traced compounds and to compare these data with currently available scientific literature and mass databases (e.g., METLIN). As a result of the compositional studies, [Supplementary-material supplementary-material-1] in the Supplementary File was sketched to present all the MS/MS spectra of the tentatively identified components.

For the qualitative determination of *R. cordatum* composition, the authors selected the richest ethanol : water (50 : 50 *v*/*v*) extracts of each organ of the plant. The LC-MS analysis of its fruits and roots showed that the tentatively identified anthraquinones in these two organs were the most abundant among all parts of the plant. The subjective quantitative analysis of each compound in every studied organ of the plant is presented in Table 1.

The plant was found to contain large quantities of emodin and its derivatives: glucoside galloyl-hexose, acetyl-hexose, or carboxyacetyl-glucoside. Beside these structures, only some traces of chrysophanol and minute quantities of aloe-emodin with its acetylated form were found. The composition of anthraquinones in this plant species is quite humble, in relation to other rhubarbs, characterized by the presence of chrysophanol, physcion, and their derivatives [25]. Contrary to the findings of McDougall and collaborators [26], the dimers of anthraquinones were not present in the studied extracts of heart-shaped rhubarbs. The analysed plant was proven to produce hexosides or acetylated forms of anthraquinones rather than dimers of aglycones.

On the other hand, the studied rhubarb can be treated as a rich source of antioxidants. It is very interesting that the phenolic portfolio is diversely represented by catechins and their gallates and by small molecules with antioxidant potential, like gallic acid, or gallic acid glucoside, and flavonoids (quercetin, apigenin, rutoside, or kaempferol). The identity of herein identified compounds is known, and these compounds have been described as present in other *Rheum* species or in *Polygonum multiflorum* (which is known as an anthraquinone-containing plant); however, according to the authors' knowledge, the majority of herein presented constituents have never been described in the scientific literature as present in this particular species—in *R. cordatum* extracts.

Compared to other rhubarbs from the same genus, the heart-shaped rhubarb contains only small quantities of resveratrol and its derivatives. The authors identified *trans-*resveratrol and tetrahydroxystilbene-*O*-(acetyl)-hexose as the stilbene derivatives present in the extracts [24, 27]. Both compounds were traced in the roots and in the stems, and their quantity was close to the methods' limit of detection in the remaining organs. Several flavonoids were present in all organs of the studied species; however, their highest concentration was found in its leaves. The analysed extracts did not contain proanthocyanidins or anthocyanins, which were represented in some other rhubarbs [26].

### 3.2. The Fingerprinting of *Rheum cordatum* Extracts

The analysed extracts differed in quantity and quality depending on the polarity of the extracting solvents used and the part of the plant tested. The extracts' fingerprints, which were obtained in the LC-MS analysis (presented in the Supplementary File Figures [Supplementary-material supplementary-material-1], [Supplementary-material supplementary-material-1], and [Supplementary-material supplementary-material-1]) showed some visible differences between the samples. On the presented graphs, the group of anthraquinones can be localised after the 25^th^ minute of analysis, whereas the phenolic compounds were eluted from the column earlier—simple phenolics at the first minutes of the analyses and flavonoid derivatives and catechins in the middle of the analyses (13-25 min). Based on [Supplementary-material supplementary-material-1] in the supplementary file, it can be easily concluded that the extractant has a leading role in the composition determination of the extracts. Dichloromethane (3) and chloroform (5) extracted only the least polar components as it could be seen in the mass chromatograms, whereas the other—more polar—metabolites were not present. [Supplementary-material supplementary-material-1] from the supplementary file shows a wide diversity of the secondary metabolites in various organs of the plant. According to the same figure, the roots and stems were the most rich in metabolites—both anthraquinones and phenolics, whereas the leaves and seeds extracts showed only several peaks in the central part of the run, which is related to the presence of flavonoids and their derivatives in these organs. Having analysed [Supplementary-material supplementary-material-1] in the supplementary file presenting the metabolites' variation in the root extracts collected in different times of the year, it could be noticed that starting from the spring until autumn, the quantity and diversity of anthraquinone derivatives decrease, contrary to flavonoids, which are more abundant and better represented in the autumn samples.

### 3.3. The Quantitative Analysis of the Most Abundant Metabolites

The authors selected seven compounds representing anthraquinones and phenolics, which were present in significant quantities in all organs to perform the quantitative analysis. This way, the authors could obtain interesting information not only on the composition of the extracts but also on the influence of extraction conditions applied (extractant selection) and the distribution of selected metabolites in the plant organs.

The quantitative analysis was performed based on the sketched calibration curves of the reference compounds in the applied analysis conditions. The obtained calibration curve equations were as follows: for isticine, *y* = 9.17888*x* − 6857142 (*R*^2^ = 0.9975); for ECG, *y* = 14449435*x* + 27462270 (*R*^2^ = 0.9982); and for rutoside, *y* = 16416641*x* + 34537978 (*R*^2^ = 0.9986). Isticine was used as an external standard for the quantification of anthraquinones, EGCG—for the quantification of catechins and rutoside for the quantification of flavonoids.

**Figure 1:**
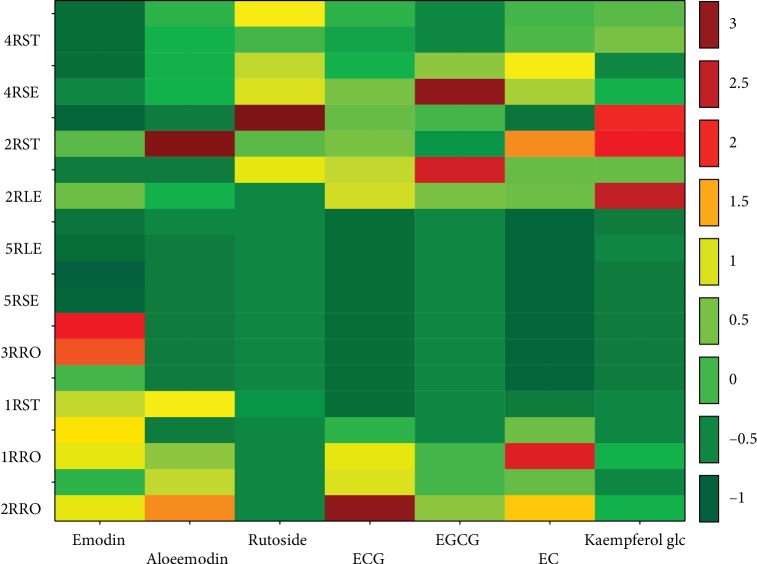
Matrix for grouping objects and features (data standardized by the *Z*-score method) (the codes applied: 1: ethanol; 2: 50% ethanol; 3: DCM; 4: H_2_O; 5: CHCl_3_: R: *Rheum*; ST: stem; SE: seed; LE: leaf; RO: root).

In the EtOH, EtOH/H_2_O, and H_2_O extracts from the leaves, stems, and seeds, the dominant compounds were rutoside (0.08-4.87%) and ECG (0.05-2.16%). However, in the extracts obtained with the same solvents from the roots, the dominant compounds were ECG (2.23-5.00%) and emodin (0.34-1.06%).  Figure 1 shows the grouping matrix of objects and features (data standardized by the *Z*-score method), and the detailed results of the below presented quantitative studies with the standard deviations of the results are shown in [Supplementary-material supplementary-material-1] of the Supplementary File.

An interestingly high variability in the concentration of emodin was demonstrated in the tested extracts. The highest content of this compound was found in the dichloromethane extract of the stem and roots: 1.17% and 1.06%, respectively. The emodin variation plot sketched in consideration of the solvent used is shown in Figure 1 and Figures [Supplementary-material supplementary-material-1] and [Supplementary-material supplementary-material-1] (Supplementary Material File). The lowest content of this compound was found in the seeds < 0.19%.

Also, the highest variability of metabolites' content expressed in the above graph was confirmed for the stem extracts (Figure 1).

### 3.4. Seasonal Variability

**Figure 2:**
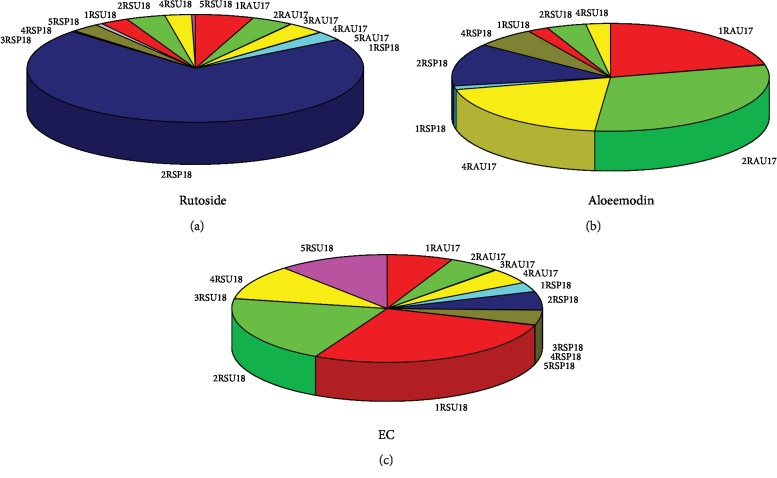
Variability of rutoside (a), aloe-emodin (b), and epicatechin (ECA) (c) contents depending on the harvesting season and the solvent used (the codes applied: 1: ethanol; 2: 50% ethanol; 3: DCM; 4: H_2_O; 5: CHCl_3_; R: *Rheum*; SU: summer—the root collected at the end of May; SP: spring—the root collected at the beginning of April; AU: autumn—the root collected in October; 18 and 17: 2018 and 2017, respectively).

The impact of the harvesting season on the content of analysed compounds in the roots showed its strong influence on the content of rutoside. It was the highest in the EtOH/H_2_O extract in the raw material harvested in the spring (0.41%) (Figure 2(a)). In the EtOH, EtOH/H_2_O, and H_2_O extracts obtained from rhubarb harvested in autumn, an increased content of aloe-emodin, 0.09%, 0.13%, and 0.08% was found, similar to its high content in the EtOH/H_2_O extract in the root harvested in spring (0.05%) (Figure 2(b)). The highest variability measured for all metabolites in the raw material harvested in the summer was found for ECA (Figure 2(c)).

### 3.5. The Antioxidant Activity of the Studied Samples

The antioxidant determinations, performed in two different models, revealed significant differences between the studied extracts. High variability of the obtained results was observed between different parts of the studied species, as well as within solvent systems used to prepare the extracts (the antioxidant activity varied from 0 to 92.8%). In general, the mixture of water and ethanol (50 : 50 *v*/*v*) was considered as the most effective solvent system to extract secondary metabolites from all parts of the plant. The average percentage of radical scavenging power for EtOH/H_2_O (50 : 50 *v*/*v*) extracts varied from 83.7% in the roots to 92.7% in its stems. However, in the case of the roots, EtOH extraction and H_2_O extraction were also efficient. The aqueous extracts showed a variable level of antioxidant activity, from 21.8% in the stem extract to 90.6% in the root extract. On the contrary, dichloromethane and chloroform were proven to be the least effective solvents, which was noticed in the results of both antioxidant tests and LC-MS determinations. In the case of EtOH/H_2_O extracts, all samples were very active, with a mean activity in the DPPH test of >90% and GAE ranging from 462 (root samples collected in April 2017) to 720 mg/L in the case of leaves. Water and pure ethanol were proven to be significantly less effective solvents for extraction of the aerial parts of *R. cordatum*: for leaves and stems, the DPPH results were <10%; for EtOH extracts, 21.8%; and for H_2_O extracts from stem and leaves, 45.9%, respectively. However, these two solvents (ethanol and water) were shown to be very effective in the extraction of underground parts—seeds and roots. For seeds, an average DPPH scavenging activity was 89.8% for the EtOH extract and 88.2% for the H_2_O extract. GAE values obtained were 460 and 470 mg/L for EtOH and H_2_O extracts, respectively. In the case of roots, it was proven that the results of antioxidant determinations were correlated with the samples' age. The younger the samples, the higher the results obtained. Among all tested root samples (year 2017, April 2018, and May 2018), the highest antioxidant potential was obtained for the water extracts from May 2018, with an average activity against DPPH free radical exceeding 90% and an average GAE value equal to 751.8 mg/L.

Results of the antioxidant activity, obtained using two different methods, were in a very good agreement. Moreover, the results of these determinations were highly correlated with LC-MS profiling of the studied extracts.

**Figure 3:**
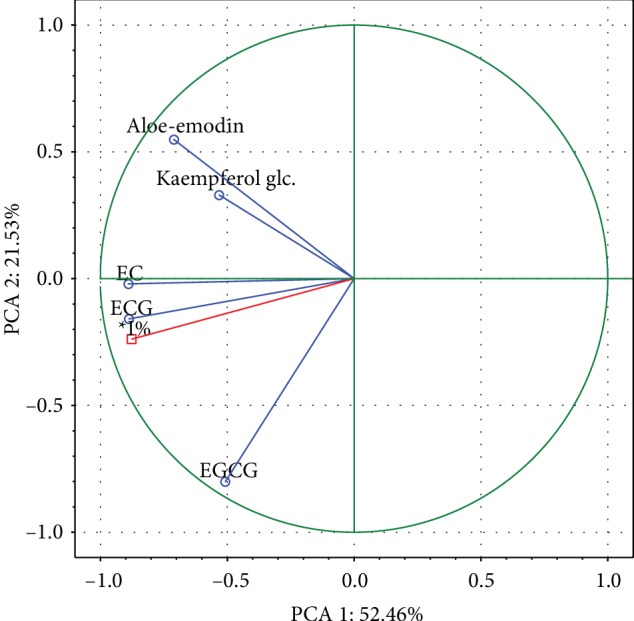
The effect of selecting the solvent on the extracted compounds presented as a projection of cases on a plane.

The antioxidant potential is affected by the chemical character and concentration of compounds. The investigated extracts showed significant correlations (*p* < 0.05) between the occurrence and content of each quantified compound. The observed antioxidant power of quantitated components can be arranged in the following order: ECA > ECG > EGCG > aloe‐emodin > kaempferol glucoside, 0.7684, 0.7660, 0.6766, 0.4866, 0.4691, respectively. The principal component analysis (PCA 1 vs. PCA 2) showed that the analysis of variance explains variation in 73.99% (Figure 3). The ECA and ECG were the most parallel to the vector indicating the per cent inhibition (% I), which indicates a good correlation between these compounds and antioxidant activity. Based on the statistical elaboration of the obtained results, it could be concluded that ECA, ECG, and EGCG are primarily metabolites, which influence the antioxidant activity of *Rheum cordatum* organs at the strongest level. In view of these findings, the plant itself may be treated as a rich source of antioxidants and might be used also for dietary purposes.

**Figure 4:**
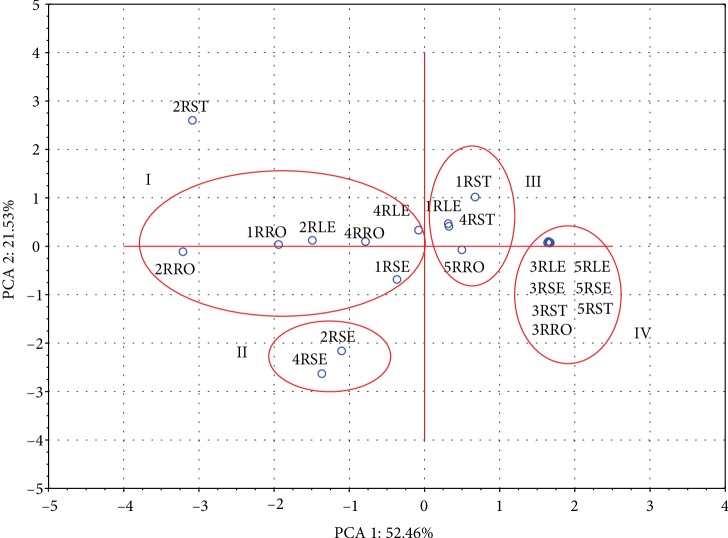
The effect of selecting the solvent on the extracted compounds was presented as a projection of cases on a plane.

The applied PCA analysis of the extracts' constituents confirmed the presence of some correlations between the extracts and their antioxidant activity (Figure 4). The EtOH/H_2_O extract from the stems (2RST) showed the highest antioxidant activity (92.7%) and was characterized by a significant content of the determined compounds. It contained the highest concentration of aloe-emodin among the tested samples.

The results of PCA 1 vs PCA 2 showed the existence of 4 clusters located in the four parts of the plane. The first cluster (I) contained the extracts with high ECA and ECG content, which simultaneously have high antioxidant activity (45.9-90.6% 4RLE-4RRO, respectively). Cluster number II was determined by the EGCG content and gathered EtOH/H_2_O and H_2_O extracts from seeds, the antioxidant activity of which was 89.1 and 88.2%, respectively. Cluster number III showed a varied content of the tested compounds with the antioxidant activity within the range of 0.0 to 21.8%. The DCM and CHCl_3_ extracts found in the fourth cluster contained small amounts of the compounds tested and showed no antioxidant activity.

## 4. Conclusions

The performed investigations shed new light on the phytochemical analysis and antioxidant activity of different organs of *Rheum cordatum*—a rare species of rhubarb, naturally present in Kazakhstan. The conducted compositional and quantitative analyses showed that the plant can be used as a strong dietary antioxidant, as they revealed that in the EtOH, EtOH/H_2_O, and H_2_O extracts from the leaves, stems, and seeds, the dominant compounds were rutoside (0.08-4.87%) and ECG (0.05-2.16%). In the extracts obtained with the same solvents from the roots, ECG (2.23-5.00%) and emodin (0.34-1.06%) were found in the highest concentrations. Significant differences were proven between different solvent systems used to extract secondary metabolites from different organs of the investigated plant. The mixture of ethanol and water (50 : 50 *v*/*v*) was found to be the most efficient in the extraction of active compounds from *R. cordatum*, whereas the dichloromethane and chloroform extracts were considered to be the least effective solvents. Quantitative determinations were in good agreement with antioxidant investigations of the studied samples. The extracts obtained with the EtOH/H_2_O mixture were found to be the most active. A marked influence of the collection season was also proven in this work. In the case of roots, the younger the samples were, the higher their antioxidant activity was. The PCA analysis of the tested samples helped to identify the secondary metabolites responsible for the antioxidant potential of the extract. Among them, catechins ECA and ECG as well as EGCG were of the highest importance.

## Figures and Tables

**Table 1:** The list of tentatively identified and traced molecules in the analysed *Rheum cordatum* ethanol : water (50 : 50 *v*/*v*) extracts together with a relative comparison of their content in the studied plant organs.

Ionisation mode	Rt (min)	Molecular formula	*m*/*z* experimental	*m*/*z* calculated	Delta (ppm)	DBE	MS/MS fragments	Tentative compound	Root	Leaf	Seed	Stem	References
*Anthraquinone derivatives*													
[M-H]^−^	24.99	C_21_H_20_O_10_	431.0971	431.0984	2.94	12	413, 269	Emodin 1-glucoside	++++	++	++	++++	[24]
[M-H]^−^	25.29	C_28_H_24_O_14_	583.1083	583.1093	1.76	17	435, 269, 169	Emodin galloyl-hexose	+	—	—	+	[27]
[M-H]^−^	25.36	C_23_H_22_O_11_	473.1084	473.1089	1.13	13	269, 186	Emodin acetyl-hexose	++	++	+	+++	[24]
[M-H]^−^	25.46	C_24_H_22_O_13_	517.1019	517.0988	-6.05	14	473, 431, 269	Emodin-8-*O*-(6′-*O*-carboxyacetyl)-*β*-D-glucoside	++	++	+	++++	[27]
[M-H]^−^	26.025	C_15_H_10_O_4_	253.0505	253.2506	0.52	11	225	Chrysophanol	+	**tr**	**tr**	+	[27]
[M-H]^−^	28.00	C_17_H_12_O_6_	311.0561	311.0561	0.04	12	269, 224, 169	Acetyl-aloe-emodin	+	—	—	+	[24]
[M-H]^−^	30.4	C_15_H_10_O_5_	269.0.454	269.0455	0.54	11	225, 180	Aloe-emodin	+	**tr**	—	+++	[27, 28]
[M-H]^−^	33.7	C_15_H_10_O_5_	269.0436	269.0455	7.21	10.5	225	Emodin	++++	++	+++	++++	[25, 27, 28]
*Phenolic compounds*													
[M-H]^−^	19.59, 24.6 and 22.67	C_28_H_24_O_14_	583.1073	583.1093	3.47	17	269, 169,	Apigenin galloyl-glucoside analogues	+	**tr**	**tr**	+	[29]
[M-H]^−^	3.55	C_7_H_6_O_5_	169.0140	169.0142	1.45	5	125	Gallic acid	++	++	+++	+	[30]
[M-H]^−^	4.75	C_13_H_16_O_10_	331.0673	331.0671	-0.69	6	169, 125	Gallic acid glucoside	++++	++++	++	+++	[24]
[M-H]^−^	13.5	C_15_H_14_O_6_	289.0698	289.0718	6.76	9	245, 203	Catechin (CA)	++	++	++	+++	[29, 31]
[M-H]^−^	16.05	C_15_H_14_O_6_	289.0699	289.0718	6.42	9	245, 205	Epicatechin (ECA)	++	+++	+++	+++	[29, 31]
[M-H]^−^	16.3	C_22_H_18_O_11_	457.0808	457.0776	-6.91	14	305, 169	Epigallocatechin gallate (EGCG)	+++	+++	++++	+++	[29, 31]
[M-H]^−^	18.58	C_21_H_20_O_10_	431.0978	431.0984	1.32	12	311, 283, 269	Apigenin glucoside	—	+++	+	++	[29, 30]
[M-H]^−^	19.0	C_22_H_18_O_10_	441.0835	441.0827	-1.76	14	289, 169	Epicatechin gallate (ECG)	++++	++++	++++	++++	[29]
[M-H]^−^	20.56	C_15_H_12_O_5_	269.0616	269.0612	-1.48	10	—	Apigenin	tr	—	—	—	[29]
[M-H]^−^	22.78	C_14_H_12_O_3_	227.0702	227.0714	5.12	9	—	*Trans*-resveratrol	+	tr	tr	**tr**	[27]
[M-H]^−^	24.2	C_15_H_10_O_7_	301.0343	301.0354	3.56	11	273, 178, 151	Quercetin	+	++++	++++	++	[31]
[M-H]^−^	24.63	C_22_H_24_O_10_	447.1289	447.1297	1.72	11	285, 242, 227	Tetrahydroxystilbene-*O*-(acetyl)-hexose	+	—	—	+	[24]
[M-H]^−^	26.9	C_15_H_10_O_6_	285.0386	285.0405	6.51	11	213, 151	Kaempferol	+	+++	+	++	[30]
[M-H]^−^	29.5	C_21_H_24_O_11_	451.1231	451.1246	3.28	10	407, 305, 179	Epicatechin glucoside	+	Tr	—	+	[29] Reference compound

DBE: double bond equivalent, delta: error of measurement in ppm; Rt: retention time; tr: traced; +, ++, +++: the relative quantity in the extracts.

## Data Availability

The data obtained within the study, that support the findings of this study are included within the article, the supplementary material files and the raw data are deposited in the Department of Pharmacognosy by the corresponding author.

## Supplementary Materials

Supplementary Materials Figure S1: compositional variation of the root extracts in relation to the collection date. Figure S2: the differences in the composition of ethanolic extracts in various organs of *Rheum cordatum* (TIC chromatograms recorded in the negative ionisation mode). Figure S3: the influence of extractant on the composition of the *Rheum cordatum* root extract (1: 98% ethanol; 2: 50% ethanol; 3: dichloromethane; 4: water; 5: chloroform extracts). Figure S4: the MS/MS spectra of the selected major constituents of the extracts. Figure S5: emodin variation graph depending on the solvent used. Figure S6: emodin variation plot depending on the part of the plant used. Table S1: content of the main constituents determined in the particular plant parts, depending on the solvents used. Table S2: the correlations between the single metabolites determined in the extracts. Table S3: the results of univariate analysis of variance. Analysis of variance. Table S4: the list of secondary metabolites present in other species of *Rheum* or *Polygonum* gender, in which the presence was not confirmed in the herein studied extracts by HR-MS studies.
