# Corrigendum to “Phytochemical and Antioxidant Studies on a Rare Rheum cordatum Losinsk: Species from Kazakhstan”

**DOI:** 10.1155/2020/9692807

**Published:** 2020-05-30

**Authors:** Gulsim Zhumashova, Wirginia Kukula-Koch, Wojciech Koch, Tomasz Baj, Galiya Sayakova, Alma Shukirbekova, Kazimierz Głowniak, Zuriyadda Sakipova

**Affiliations:** ^1^School of Pharmacy, S.D. Asfendiyarov Kazakh, National Medical University, Tole-bi 94, 050012 Almaty, Kazakhstan; ^2^Department of Pharmacognosy, Medical University of Lublin, 1 Chodzki Str., 20-093 Lublin, Poland; ^3^Department of Food and Nutrition, Medical University of Lublin, 4a Chodzki Str., 20-093 Lublin, Poland; ^4^Department of Pharmaceutical Disciplines, Astana Medical University, Beybitshilik 49A, 010000 Nur-Sultan, Kazakhstan; ^5^Department of Cosmetology, University of Information Technology and Management in Rzeszów, Kielnarowa 386a, 36-020 Tyczyn, Poland

In the article titled “Phytochemical and Antioxidant Studies on a Rare Rheum cordatum Losinsk: Species from Kazakhstan” [1], author Tomasz Baj was affiliated to affiliation 1 in error. The correct affiliation for this author is shown below and has been updated in the author information above.

Department of Pharmacognosy, Medical University of Lublin, 1 Chodzki Str., 20-093 Lublin, Poland.
