# Supplementary material for: Phytochemical and Antioxidant Studies on a Rare Rheum cordatum Losinsk. Species from Kazakhstan
Source: Oxid Med Cell Longev. 2019 Nov 16;2019:5465463. doi: 10.1155/2019/5465463 (PMC6885188; doi:10.1155/2019/5465463)
Supplement: Supplementary Materials — Figure S1: compositional variation of the root extracts in relation to the collection date. Figure S2: the differences in the composition of ethanolic extracts in various organs of Rheum cordatum (TIC chromatograms recorded in the negative ionisation mode). Figure S3: the influence of extractant on the composition of the Rheum cordatum root extract (1: 98% ethanol; 2: 50% ethanol; 3: dichloromethane; 4: water; 5: chloroform extracts). Figure S4: the MS/MS spectra of the selected major constituents of the extracts. Figure S5: emodin variation graph depending on the solvent used. Figure S6: emodin variation plot depending on the part of the plant used. Table S1: content of the main constituents determined in the particular plant parts, depending on the solvents used. Table S2: the correlations between the single metabolites determined in the extracts. Table S3: the results of univariate analysis of variance. Analysis of variance. Table S4: the list of secondary metabolites present in other species of Rheum or Polygonum gender, in which the presence was not confirmed in the herein studied extracts by HR-MS studies. [file 5465463.f1.docx]

**Supplementary Material**

# Phytochemical and antioxidant studies on a rare *Rheum cordatum* Losinsk. species from Kazakhstan

Gulsim Zhumashova, Wirginia Kukula-Koch,^*^ Wojciech Koch, Tomasz Baj, Galiya Sayakova, Alma Shukirbekova, Kazimierz Głowniak and Zuriyadda Sakipova^1^

**Figure S1. Compositional variation of the root extracts in relation to the collection date**

**
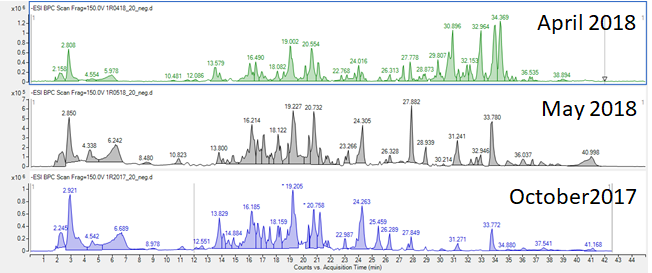
**

**Figure S2. The differences in the composition of ethanolic extracts in various organs of *Rheum cordatum* (TIC chromatograms recorded in the negative ionisation mode)**

***
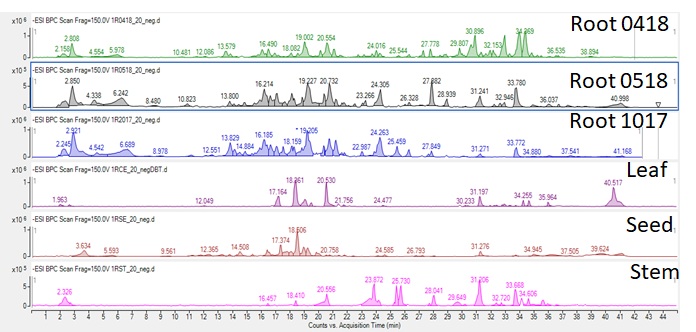
***

**Figure S3**. The influence of extractant on the composition of the *Rheum cordatum* root extract (1- 98% ethanol, 2- 50% ethanol, 3-dichloromethane, 4-water, 5-chloroform extracts)


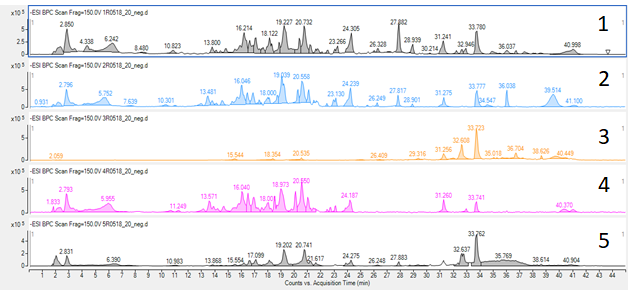


**Figure S4**. The MS/MS spectra of the selected major constituents of the extracts

| Acetyl aloe-emodin**** | Aloeemodin |
| --- | --- |
| Apigenin galloyl glucose | Apigenin glucoside |
| Catechin | Chrysophanol**** |
| Emodin | Emodin acetyl hexose |
| Emodin galloylglucose**** | Emodin glucoside |
| Emodin malonyl hexose | Epicatechin |
| Epicatechin gallate | Epigallocatechin gallate |
| Gallic acid | Gallic acid hexoside |
| Kaempferol | Quercetin**** |
| Tetrahydroxystilbene-O-(acetyl)-hexose**** |  |

Table S1: Content of the main constituents determined in the particular plant parts, depending on the solvents used.

|  |  | Leaves | Seeds | stems | Radix 2017 | Radix  04.2018 | Radix 05.2018 |
| --- | --- | --- | --- | --- | --- | --- | --- |
| **Ethanol extract** | Emodin | 0.0614±0.0009 | 0.0694±0.0017 | 0.6581±0.0056 | 0.5174±0.0043 | 0.3626±0.003 | 0.8023±0.0152 |
|  | Aloeemodin | 0.0037±0.00006 | 0.0029±0.00002 | 0.0135±0.00008 | 0.0889±0.0021 | 0.0049±0.0007 | 0.0089±0.0001 |
|  | ECG | 0.8552±0.0264 | 0.7824±0.0147 | 0.0509±0.0011 | 2.93±0.0921 | 2.5052±0.0276 | 2.38±0.0316 |
|  | EGCG | 0.0151±0.0002 | 0.24±0.0064 | 0.0001±0.000003 | 0.4394±0.0034 | 0.2467±0.0035 | 0.1265±0.005 |
|  | EC | 0.0304±0.0009 | 0.0736±  0.0027 | 0.0116±0.00008 | 0.0274±0.0012 | 0.0114±0.0002 | 0.1100±0.0042 |
|  | Rutoside | 2.108±0.0244 | 1.614±0.0361 | 0.1504±0.0026 | 0.0327±0.0002 | 0.0155±0.0003 | 0.0160±0.0003 |
|  | Kaempferol glucoside | 0.0410±0.0004 | 0.0032±0.0001 | 0.0043±0.00006 | 0.0433±0.001 | 0.0428±0.0006 | 0.0225±0.0001 |
|  | DPPH | <LOQ | 89.80±1.97 | <LOQ | 92.05±0.39 | 92.3±0.69 | 68.13±4.18 |
|  | TPC | <LOQ | 460±24.70 | <LOQ | 530.2±37.65 | 430.2±28.67 | 720.3±34.22 |
| **Ethanol:water** | Emodin | 0.5420±0.0114 | 0.1416±0.0012 | 0.4482±0.0194 | 1.29±0.0068 | 0.9650±0.027 | 0.7956±0.022 |
|  | Aloeemodin | 0.0025±0.00002 | <LOQ | 0.0293±0.0003 | 0.1273±0.0007 | 0.0539±0.0036 | 0.017±0.0003 |
|  | ECG | 2.1589±0.0131 | 2.0829±0.0031 | 1.6876±0.0132 | 3.084±0.0319 | 3.3669±0.0262 | 5.004±0.1265 |
|  | EGCG | 0.2350±0.0017 | 0.6432±0.0195 | 0.0363±0.0003 | 0.2212±0.0028 | 0.3596±0.0026 | 0.24±0.0015 |
|  | EC | 0.0464±0.0005 | 0.0416±0.0004 | 0.0873±0.005 | 0.0213±0.00004 | 0.0222±0.0005 | 0.081±0.0033 |
|  | Rutoside | 0.0757±0.0009 | 2.0235±0.0106 | 0.8525±0.009 | 0.0232±0.00003 | 0.4144±0.0064 | 0.022±0.0005 |
|  | Kaempferol glucoside | 0.1564±0.0051 | 0.0466±0.0001 | 0.1408±0.0021 | 0.0549±0.0017 | 0.0159±0.0007 | 0.021±0.0003 |
|  | DPPH | 89.96±0.4559 | 89.06±1.54 | 92.74±0.9577 | 88.48±0.68 | 89.69±1.63 | 83.7±2.08 |
|  | TPC | 719.8±26.61 | 669.8±19.86 | 540.2±30.74 | 480.3±30.07 | 462±23.56 | 620.7±39.93 |
| **Dichloromethene** | Emodin | 0.1241±0.0005 | 0.0004±0.00001 | 1.1747±0.0354 | 0.2855±0.0009 | 0.7823±0.0180 | 1.0646±0.0287 |
|  | Aloeemodin | 0.0002±0.00002 | <LOQ | <LOQ | <LOQ | <LOQ | <LOQ |
|  | ECG | 0.0032±0.0002 | 0.0022±0.00002 | 0.0403±0.0021 | 0.0019±0.00004 | 0.0045±0.00009 | 0.0014±0.00015 |
|  | EGCG | <LOQ | 0.0003±0.00002 | 0.0008±0.00005 | 0.0002±0.000006 | 0.0003±0.00002 | 0.00009±0.00001 |
|  | EC | 0.0007±0.00002 | 0.0002±0.000002 | 0.0006±0.00004 | 0.0004±0.00003 | 0.0003±0.00002 | 0.0003±0.00002 |
|  | Rutoside | 0.0106±0.0002 | 0.0014±0.00005 | 0.0059±0.0001 | 0.0003±0.00003 | 0.0018±0.00001 | <LOQ |
|  | Kaempferol glucoside | <LOQ | 0.0005±0.00001 | 0.0019±0.00006 | <LOQ | <LOQ | <LOQ |
|  | DPPH | <LOQ | <LOQ | <LOQ | <LOQ | <LOQ | <LOQ |
|  | TPC | <LOQ | <LOQ | <LOQ | <LOQ | <LOQ | <LOQ |
| **Water** | Emodin | 0.0197±0.0001 | 0.1898±0.001 | 0.06±0.0008 | 0.8431±0.0308 | 0.5222±0.0337 | 0.3369±0.011 |
|  | Aloeemodin | <LOQ | 0.0029±0.000009 | 0.0027±0.00005 | 0.0831±0.001 | 0.0257±0.0028 | 0.011±0.00008 |
|  | ECG | 1.424±0.0111 | 1.7212±  0.0035 | 0.6364±0.0134 | 2.5042±0.0131 | 2.76±0.1116 | 2.29±0.0196 |
|  | EGCG | 0.1107±0.0005 | 0.7922±0.0097 | 0.0144±0.00004 | 0.1564±0.0004 | 0.2335±0.0014 | 0.1224±0.0028 |
|  | EC | 0.0087±0.00004 | 0.0557±0.0021 | 0.0341±0.0002 | 0.0186±0.00009 | 0.0178±0.0003 | 0.0421±0.0009 |
|  | Rutoside | 4.8664±0.0604 | 1.9050±0.0102 | 0.5992±0.0171 | 0.0211±0.0003 | 0.0157±0.0006 | 0.015±0.0004 |
|  | Kaempferol glucoside | 0.1299±0.0016 | 0.0181±0.00005 | 0.0567±0.002 | 0.014±0.00006 | 0.011±0.0013 | 0.0049±0.00007 |
|  | DPPH | 45.9±1.38 | 88.23±1.26 | 21.83±8.42 | 82.79±2.07 | 91.59±1.11 | 90.6±1.00 |
|  | TPC | 279.8±37.35 | 470.3±18.75 | 90.33±9.52 | 320±31.55 | 498±19.71 | 751.8±34.78 |
| **Chloroform** | Emodin | 0.0658±0.0026 | 0.0375±0.0006 | 0.37±0.0038 | 0.5656±0.0417 | 0.6566±0.0161 | 0.8611±0.0195 |
|  | Aloeemodin | <LOQ | <LOQ | <LOQ | <LOQ | <LOQ | <LOQ |
|  | ECG | 0.0024±0.00002 | 0.0019±0.00002 | 0.0035±0.00005 | 0.0034±0.00005 | 0.0011±0.00005 | 0.8647±0.0545 |
|  | EGCG | 0.0001±0.000005 | 0.0002±0.000001 | 0.0002±0.00001 | 0.0006±0.00004 | 0.0002±0.00002 | 0.0193±0.00005 |
|  | EC | 0.0001±0.00002 | 0.0008±0.00003 | 0.0006±0.0001 | <LOQ | 0.0003±0.00003 | 0.0459±0.0015 |
|  | Rutoside | 0.0007±0.0001 | 0.0005±0.00001 | 0.0005±0.00006 | 0.0004±0.00001 | 0.0039±0.0002 | 0.0023±0.00005 |
|  | Kaempferol glucoside | 0.0041±0.00003 | <LOQ | <LOQ | 0.0042±0.00008 | 0.0102±0.0007 | 0.0076±0.0003 |
|  | DPPH | <LOQ | <LOQ | <LOQ | <LOQ | 21.71±7.36 | <LOQ |
|  | TPC | <LOQ | <LOQ | <LOQ | <LOQ | <LOQ | <LOQ |

**Table S2.** The correlations between the single metabolites determined in the extracts

|  | *I%* | *Emodin* | *Aloeemodin* | *ECG* | *EGCG* | *EC* | *Rutoside* | *Kaempferol glucoside* |
| --- | --- | --- | --- | --- | --- | --- | --- | --- |
| I% | 1 |  |  |  |  |  |  |  |
| Emodin | -0.0173 | 1 |  |  |  |  |  |  |
| Aloeemodin | 0.3448 | 0.259271 | 1 |  |  |  |  |  |
| ECG | 0.8852 | -0.05801 | 0.256912 | 1 |  |  |  |  |
| EGCG | 0.7165 | -0.12694 | -0.11936 | 0.695647 | 1 |  |  |  |
| EC | 0.8673 | -0.03761 | 0.589719 | 0.721823 | 0.48493 | 1 |  |  |
| Rutoside | 0.3956 | -0.35367 | -0.05216 | 0.53824 | 0.385864 | 0.256191 | 1 |  |
| Kaempferol glucoside | 0.5809 | 0.048616 | 0.424163 | 0.759295 | 0.094772 | 0.489354 | 0.415671 | 1 |

**Table S3.** The results of univariate analysis of variance

| *Groups* | *Counter* | *Sum* | *Average* | *Variance* |  |  |
| --- | --- | --- | --- | --- | --- | --- |
| Emodin | 15 | 3.962698 | 0.26418 | 0.10624 |  |  |
| Aloeemodin | 15 | 0.0578 | 0.003853 | 6.15E-05 |  |  |
| ECG | 15 | 11.45336 | 0.763558 | 0.703273 |  |  |
| EGCG | 15 | 2.088774 | 0.139252 | 0.062664 |  |  |
| EC | 15 | 0.392295 | 0.026153 | 0.000861 |  |  |
| Rutoside | 15 | 14.21484 | 0.947656 | 1.864206 |  |  |
| Kaempferol glucoside | 15 | 0.603319 | 0.040221 | 0.003165 |  |  |
|  |  |  |  |  |  |  |
|  |  |  |  |  |  |  |
| ANALYSIS OF VARIANCE | |  |  |  |  |  |
| *Source of variance* | *SS* | *df* | *MS* | *F* | *p value* | *Test F* |
| between  groups | 13.35927 | 6 | 2.226544 | 5.687277 | 4.18242E-05 | 2.192518 |
| inside groups | 38.36657 | 98 | 0.391496 |  |  |  |
|  |  |  |  |  |  |  |
| Sum | 51.72584 | 104 |  |  |  |  |

**Figure S5.** Emodin variation graph depending on the solvent used


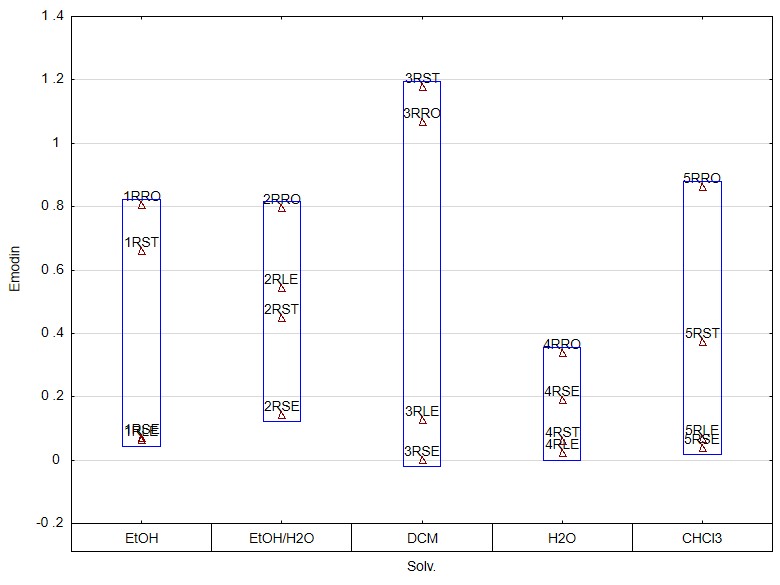


**Figure S6**. Emodin variation plot depending on the part of the plant used.


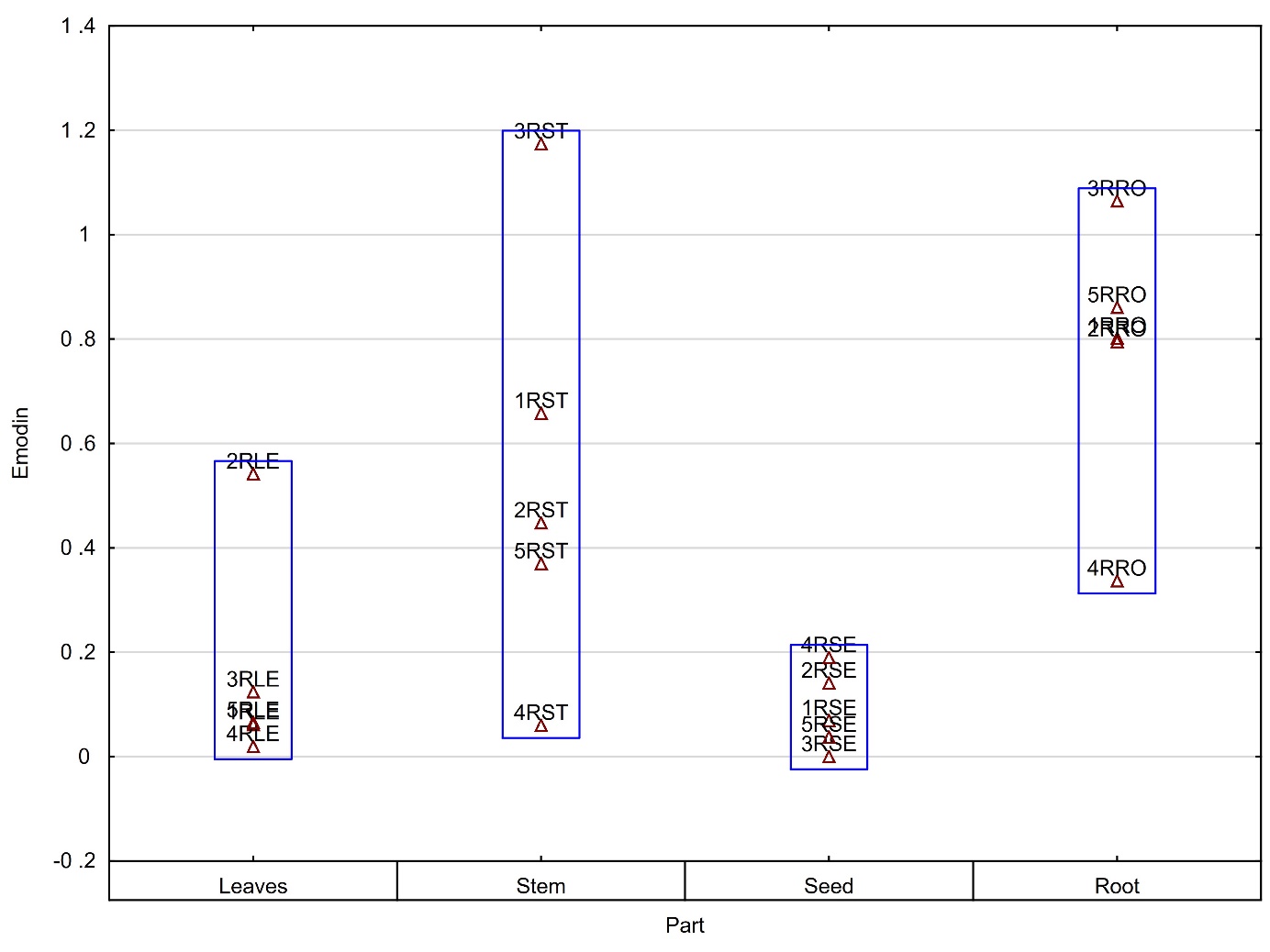


**Table S4.** The list of secondary metabolites present in other species of *Rheum* or *Polygonum* gender, **which presence was not confirmed** in the herein studied extracts by HR-MS studies.

| **Name** | **Name** |
| --- | --- |
| Physcion | Gentisin |
| Rhein | Aloeemodin acetyl-hexoside |
| Chrysophanol hexoside | Hydroxyemodin |
| Dimer of rhein and emodin and acetyl-hexose | Rhapontigen hexoside |
| Rhein | Resveratrol glucoside |
| Rhein hexoside | Emodin malonyl-glucoside |
| Chrysophanol acetyl-hexoside | Dimer of emodin and chrysophanol |
